# Survival analysis of a 16-year cohort of follicular lymphoma patients receiving systemic treatment in Brazil

**DOI:** 10.3389/fphar.2024.1414244

**Published:** 2025-01-10

**Authors:** Pamela Santos Azevedo, Isabella Zuppo Laper, Deborah Marta do Santos Oliveira, Adriano de Paula Sabino, Marina Morgado Garcia, Isabela Cristina Menezes de Freitas, Wallace Mateus Prata, Mariângela Leal Cherchiglia, Juliana Álvares-Teodoro, Francisco de Assis Acurcio, Augusto Afonso Guerra Júnior

**Affiliations:** ^1^ Department of Social Pharmacy, Federal University of Minas Gerais, Belo Horizonte, Brazil; ^2^ Department of Clinical and Toxicological Analyses, Federal University of Minas Gerais, Belo Horizonte, Brazil; ^3^ Collaborating Centre for Health Technology Assessment and Excellence (CCATES), Belo Horizonte, Brazil; ^4^ Research and Development Directorate of the Ezequiel Dias Foundation, Belo Horizonte, Minas Gerais, Brazil; ^5^ Department of Preventive and Social Medicine at the Federal University of Minas Gerais, Belo Horizonte, Minas Gerais, Brazil

**Keywords:** follicular lymphoma, rituximab, survival analysis, real-world evidence, brazilian unified health system

## Abstract

**Introduction:**

Follicular lymphoma (FL) is a common type of non-Hodgkin lymphoma that is incurable but often follows an indolent course. While survival is improving thanks to advances in diagnosis, supportive care, and new therapies, understanding outcomes and their impact on overall survival is still limited. There are few studies on FL in Brazil, so this study aims to evaluate the patient’s profile, morbidity and mortality treated by the Brazilian national health service (SUS) and evaluate risk factors associated with treatment failure.

**Methods:**

This is a nationwide 16 years cohort with patients that underwent chemotherapy in the SUS (2000–2015). The Kaplan-Meier method was used to estimate survival until treatment failure, and the Cox proportional hazards model was used to evaluate risk factors.

**Results:**

The cohort included 10,009 patients and survival rates were 73.3%, 45.3%, and 30.7% for the first, fifth and 10th year respectively. The median overall survival was approximately 4.1 years. The most used regimen was CHOP (13%), followed by CVP (9.7%) and R-CHOP (3.3%). Four hundred and ninety-eight patients (4.9%) used rituximab-containing regimens. Univariate analysis indicated worse survival rates for male patients, those over 65 years of age, clinical stage III or IV and those using non-rituximab-containing regimens. The health technology performance assessment related to oncology schemes for FL suggests that rituximab-based regimens has shown best survival probability (0.52 CI 0.39–0.69) in 78 months of follow up with a HR 1.5 times better than other schemes (HR 0.67; CI 0.55–0.81).

**Discussion:**

In light of the substantial advancements achieved by the SUS, there is a need for CONITEC to expedite decision-making processes in order to enhance patients access to new oncology drugs. This should be done while upholding health technology assessment standards. Timely integration and sufficient funding for oncology services have the potential to save lives, especially when compared to the treatments available within SUS at that time.

## 1 Introduction

Follicular lymphoma (FL) is one of the most common types of indolent non-Hodgkin lymphoma (NHL) in the Western hemisphere. It comprises approximately 22% of all cases of NHL and is primarily derived from B cells. While the exact cause of FL is often unknown, factors such as heredity, immunological disorders, viral infections, and environmental, occupational, and dietary exposures may be related to its development. NHL is more common in males, and the risk increases with age (Armitage et al., 2017; Swerdlow et al., 2016).

The Brazilian National Cancer Institute (INCA) estimates thousands of new NHL cases annually. Specific epidemiological data for FL are limited. Treatment options in the Brazilian Unified Health System (SUS) are directed by specific guidelines, and they may include radiation therapy, chemotherapy, immunotherapy, and hematopoietic stem cell transplantation. Medication-based treatment, such as the CHOP regimen, is typically the first choice, with rituximab added in some cases. Despite FL being incurable, overall patient survival continues to improve due to advancements in diagnosis, supportive care, and the development of new therapies. Real-world evidence plays a crucial role in clinical decision-making and healthcare policy formulation. Utilizing such evidence can efficiently answer questions and provide valuable insights into patient outcomes and healthcare system effectiveness (INCA, 2023).

In Brazil, the public health system provides access to various databases managed by the Department of Health Informatics (DATASUS), which track epidemiological data. These databases include the Hospital Information System (SIH/SUS), Mortality Information System (SIM/SUS), and Ambulatory Information System (SIA/SUS). These data sources are essential for health research. Currently, there is no study in Brazil that assesses the survival probability and associated risk factors of death for FL patients who received outpatient and/or inpatient oncological treatment through SUS data. Conducting a real-world data study has the potential to inform both clinical decisions and healthcare policy formulation, contributing to the sustainability and efficiency of the healthcare system by representing the real impact of treatments on patients' lives (Departamento de Informática em Saúde, 2013; Brasil. Ministério Da Saúde (MS), 2014).

## 2 Methods

We conducted a retrospective cohort study of patients who underwent hospital and outpatient clinical related to FL treatment in SUS from 01/01/2000 to 12/31/2015. A National Database of Health centered on the individual was built through a deterministic-probabilistic record linkage of three administrative databases: The Outpatient Information System (SIA/SUS), the Hospital Information System (SIH/SUS), and the Mortality Information System (SIM). The construction and validation of this database has been described and validated elsewhere, and it has been used in previous research studies published (Paulino et al., 2020; Lemos et al., 2018). The period of the cohort was determined based on the data available within the database (Lemos et al., 2018; Acurcio et al., 2020; Guerra-Júnior et al., 2017).

The study included patients with FL according to the 10th revision of the international classification of diseases (ICD-10) who had an oncological diagnosis identified as C82, C821, C822, C827 and C829 (which are the classifications presented in the SUS treatment guideline), who underwent the following procedures.

• Chemotherapy for low-grade non-Hodgkin lymphoma (first Line).
• Chemotherapy for intermediate or high-grade non-Hodgkin lymphoma (first Line).
• Chemotherapy for low-grade non-Hodgkin lymphoma (second Line).
• Chemotherapy for intermediate or high-grade non-Hodgkin lymphoma (second Line).
• Chemotherapy for intermediate or high-grade non-Hodgkin lymphoma (third Line).

The date of the first procedure of chemotherapy with the ICD group C82 was defined as the cohort entry date. The follow-up period spanned from January 2000 to December 2015 (16 years of follow-up), with the cohort entry cut-off date set as the date of the first procedure recorded until December 2014. This ensured a minimum follow-up of 12 months (1 year). Additionally, the analysis focused on new users, meaning only patients who initiated treatment on or after 1 January 2000, were included, and patients with cohort entry declared before the year 2000 were excluded. All patients were followed until the end of the study (right censored).

We analyzed the factors that influenced survival using univariate analysis for each descriptive variable and evaluated their association with the event—death. The Kaplan-Meier method was used to estimate the cumulative probability of survival. The different survival curves were compared using the log rank test. The hazard ratio (HR) for progression to the event was calculated by univariate analyses considering a 95% confidence interval (95% CI), using the Cox proportional hazards model and comparing each variable with the values of the same group.

The following variables were analyzed: sex, age at diagnosis, self-declared skin color, country´s region, diagnosis’ year, clinical stage at diagnosis (Stage I: tumor is small and low grade -GX or G1; Stage II: tumor is small and G2 or G3; Stage III: Tumor is larger and G2 or G3; and Stage IV: cancer has spread to other parts of the body), (any G)), first course-therapy, frailty index, comorbidity Charlson index and time of death.

Considering the possibility of potential bias from including patients with aggressive lymphoma in the cohort, we performed a sensitivity analysis, excluding patients with this diagnosis.

### 2.1 Statistical analysis

Descriptive statistical analysis of all variables in this study was performed: frequency distribution for categorical variables and central tendency for continuous variables.

The Kaplan-Meier method was used to estimate the cumulative probability of survival and different survival curves were compared using the log-rank test. The hazard-ratio (HR) for progression to the event was calculated by univariate analysis considering a 95% confidence interval.

The management of the data and statistical analysis was performed using ‘R’ Version 4.3.1 (R Foundation for Statistical Computing) and MySQL,19 version 5.0(Oracle Corporation), considering a significance level of 5%.

### 2.2 Ethical aspects

The use of the National Database was evaluated by the Research Ethics Committee of the Federal University of Minas Gerais, which provided a favorable opinion (CAAE - 16334413.9.0000.5149). The research was conducted in accordance with all relevant Brazilian guidelines and regulations and followed the criteria of the Declaration of Helsinki.

## 3 Results

### 3.1 Demographic characteristics

**FIGURE 1:**
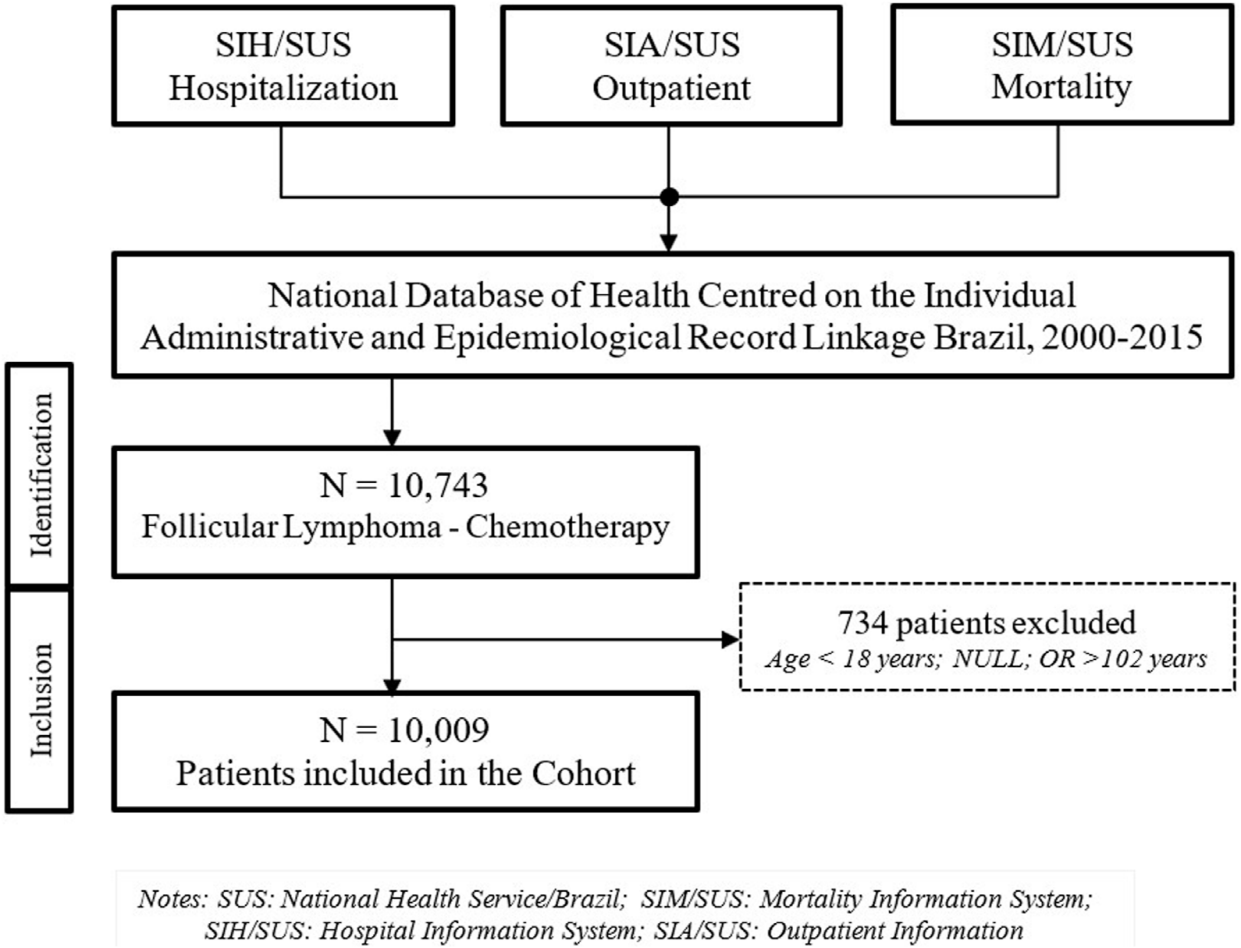
Flowchart for patient inclusion and exclusion in the cohort..

A total of 10,743 patients with FL were identified in the National Database of Health Centred on the Individual with Administrative and Epidemiological Record Linkage Brazil used in this analysis from the year 2000–2015 (Junior et al., 2018). These databases contain information from national health services across the 26 Brazilian states plus the Federal District. This cohort included patients who underwent at least one of the procedures defined as inclusion criteria for FL. However, 734 (6.8%) patients were excluded from the cohort because they were either under 18 or over 102 years of age at the start of treatment or lacked age-related data. In the end, 10,009 FL patients with available data were included in the analysis (Figure 1).

The average age of the analyzed patients was 55 years, with 29% of them being over 65 years old. Fifty-three percent of the cohort (5,292) were male. Most patients (3,513, accounting for 35%) underwent their first procedure for FL treatment between 2008 and 2011. Additionally, there was underreporting of tumor grade and clinical staging, with GX (undefined) and indeterminate clinical stage being the most common entries for these variables, at 79% and 38%, respectively. Most patients resided in the Southeast (48%) and South (27%) regions, followed by the Northeast, Midwest, and North regions (18%, 4.5%, 2.3%, respectively). Other epidemiological characteristics are summarized in Table 1.

**TABLE 1:** Epidemiological characteristics of follicular lymphoma’s patients.

Patients Characteristics	N = 10,009
Sex	
Male	5,292 (53%)
Female	4,717 (47%)
Age at baseline	55 (16)
Age range at baseline	
>65 years	2,919 (29%)
56–65 years	2,456 (25%)
46–55 years	2,048 (20%)
36–45 years	1,234 (12%)
26–35 years	822 (8.2%)
18–25 years	530 (5.3%)
Self-declared skin color	
White	3,748 (37%)
Non-Caucasian (Mixed)	1,140 (11%)
Black	162 (1.6%)
Asian	75 (0.7%)
Indigenous	4 (<0.1%)
Unknown	4,880 (49%)
Residence region at baseline	
Southeast	4,810 (48%)
South	2,687 (27%)
Northeast	1,824 (18%)
Midwest	452 (4.5%)
North	235 (2.3%)
Unknown	1 (0.01%)
Cohort entry period	
2008–2011	3,513 (35%)
2012–2015	2,331 (23%)
2000–2003	2,092 (21%)
2004–2007	2,073 (21%)
Diagnose ICD10 at baseline	
C82-Follicular non-Hodgkin’s lymphoma	49 (0.5%)
C820-Non-Hodgkin’s lymphoma, small cleaved cells, follicular	2,865 (29%)
C821-Non-Hodgkin’s lymphoma, mixed, small and large cleaved cells, follicular	641 (6.4%)
C822-Non-Hodgkin’s lymphoma, large cell, follicular	1,900 (19%)
C827-Other types of non-Hodgkin lymphoma, follicular	792 (7.9%)
C829-Non-Hodgkin’s lymphoma, follicular, unspecified	3,762 (38%)
C82-Follicular non-Hodgkin’s lymphoma	49 (0.5%)
Clinical stage at baseline	
I	772 (7.7%)
II	1,344 (13%)
III	1,942 (19%)
IV	2,158 (22%)
Unknown	3,793 (38%)
Tumor grade at baseline	
G1	1,496 (15%)
G3	573 (5.7%)
G2	4 (<0.1%)
GX	7,936 (79%)
Chemotherapy at baseline	
Other Schemes	3,447 (34%)
Rituximab based	498 (5.0%)
Unknown*	6,064 (61%)
Frailty index at baseline	13 (150)
Comorbidity Charlson Index at baseline	4 (3)
Time in the cohort	31 (36)
Event type	
Censuring	5,540 (55%)
Death	4,469 (45%)

*(Armitage et al., 2017) n (%); Mean (SD).

Therapeutic schemes from years 2000–2007, are not registered in the databases.

Although histopathological and molecular data are not available in the database, it was possible to identify a change in the ICD for patients after undergoing procedures related to FL. Out of a total of 10,009 patients, 1,431 had their ICD modified during treatment to a more aggressive non-Hodgkin lymphoma, such as diffuse large B-cell lymphoma, after entering the cohort.

**FIGURE 2:**
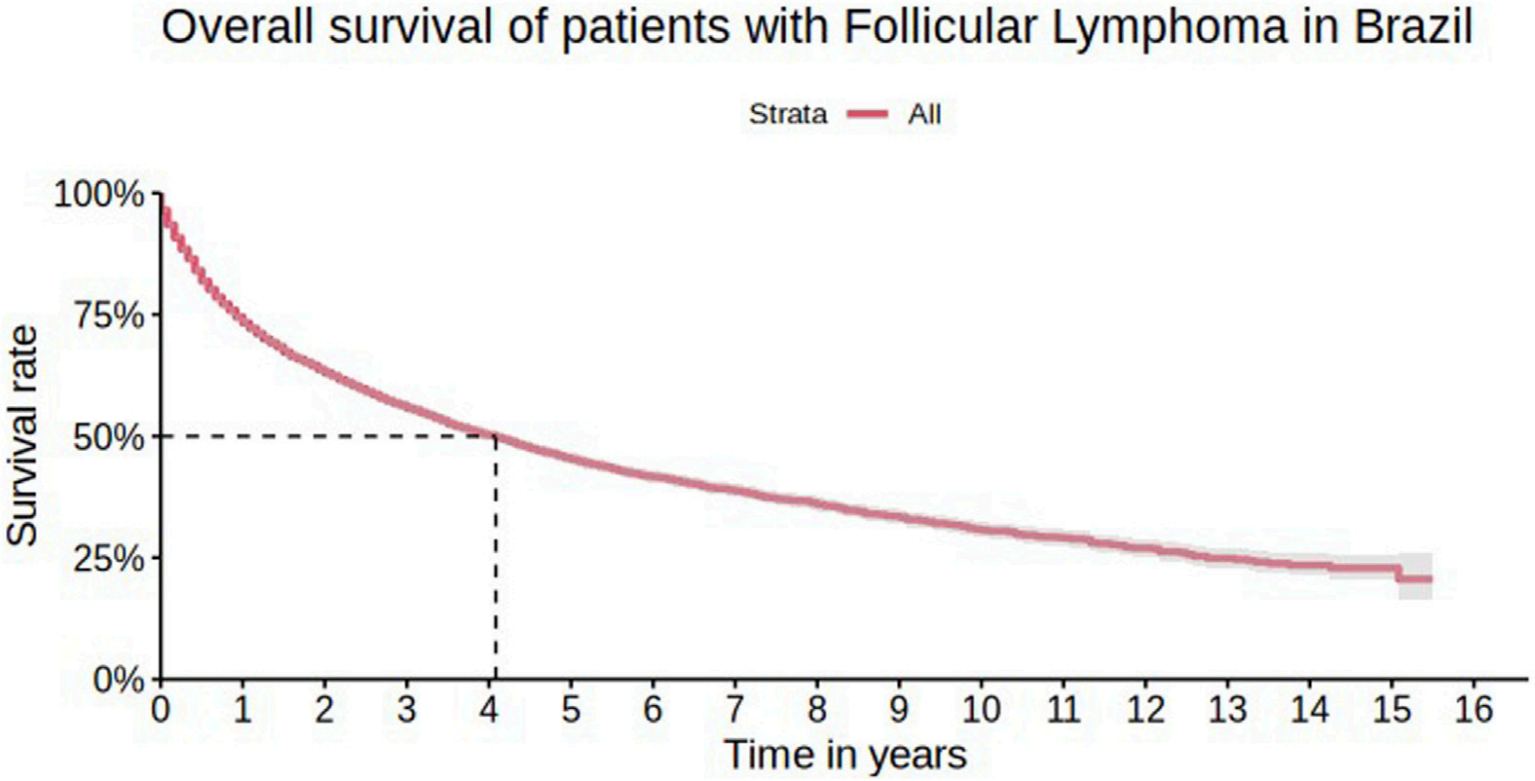
Overall survival of patients with follicular lymphoma in Brazil.

During the follow-up period for the 10,009 patients, there were 4,469 events in the cohort. The estimated overall survival (OS) at one, five, and 10 years of follow-up was 73.3% (95% CI 72.3%–74.2%), 45.3% (95% CI 44.0%–46.5%), and 30.7% (95% CI 29.1%–32.4%), respectively. The median OS was 49 months (95% CI 49–46 months), approximately 4.1 years, as shown in Figure 2.

**FIGURE 3:**
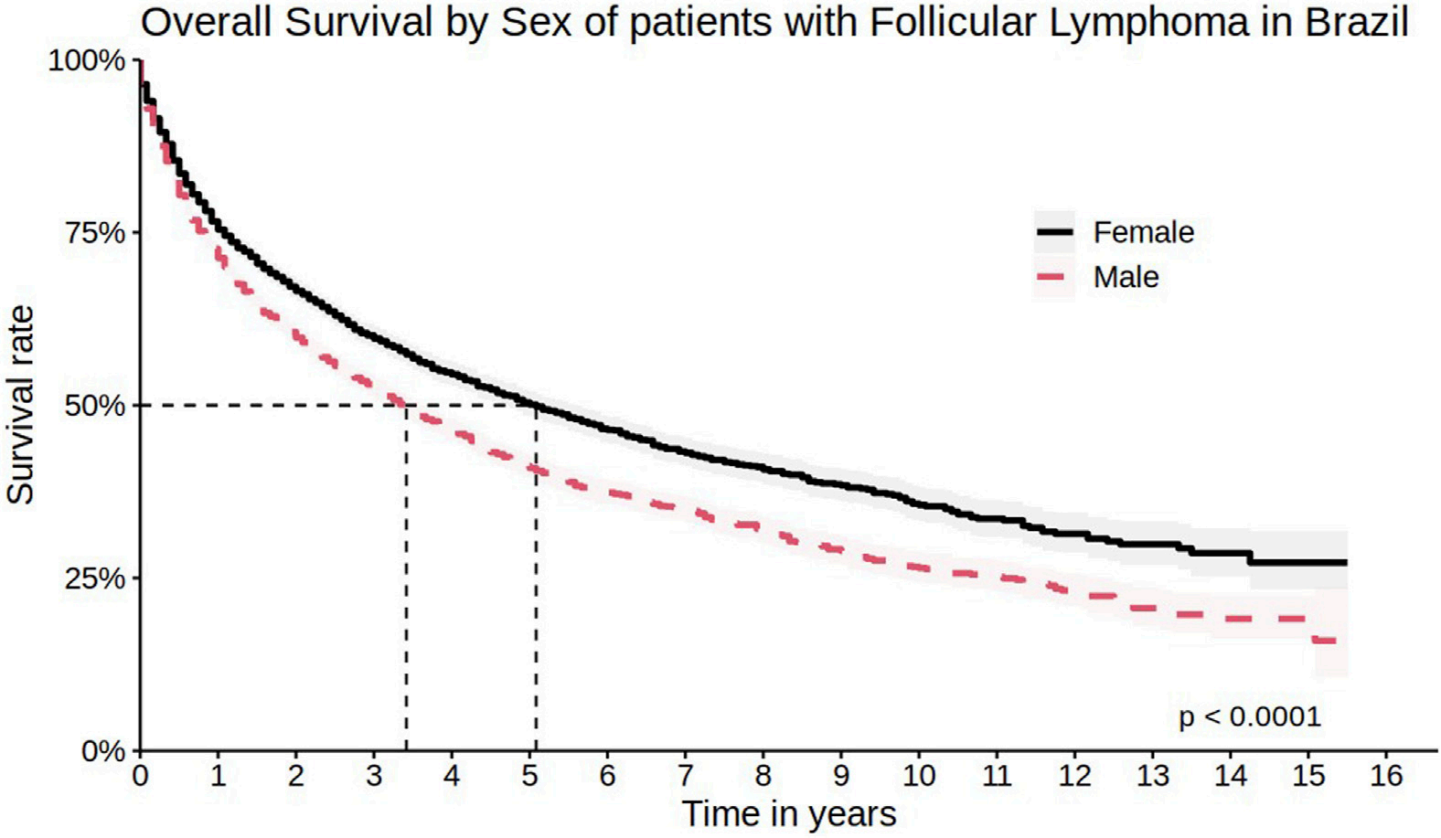
Overall survival by sex of patients with follicular lymphoma in Brazil.

**FIGURE 4:**
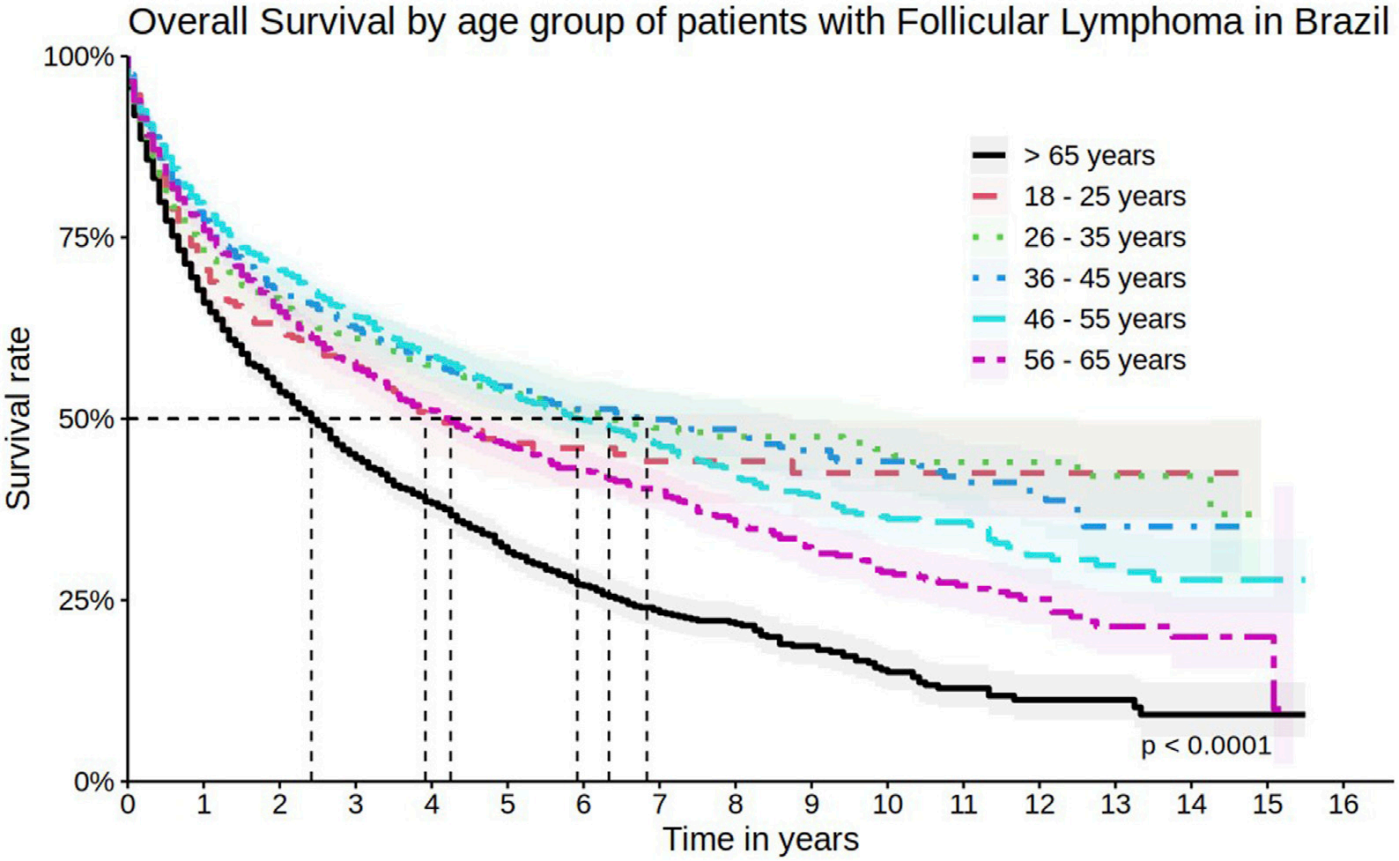
Overall survival by age group of patients with follicular lymphoma in Brazil.

The survival rate was lower among men, with 1-year, 5-year, and 10-year survival rates of 71.3% (95% CI 70.0%–72.6%), 40.9% (95% CI 39.2%–42.7%), and 26.5% (95% CI 24.4%–28.7%), respectively (Figure 3), as well as in elderly patients (>65 years old) with 1-year and 5-year survival rates of 65.9% (95% CI 64.2%–67.8%) and 31.6% (95% CI 29.4%–34.0%), respectively (Figure 4).

The following characteristics were associated with lower survival rates in the univariate analysis of the cohort: male gender, age above 65 years, clinical stage III, IV, and indeterminate at the time of first treatment, patients entering the cohort between 2000 and 2011, frailty index and comorbidity Charlson index. (Table 2).

**TABLE 2:** Univariate analysis of the cohort: Impact of patient characteristics on survival.

Patients Characteristics	HR	95% CI	*p*-value
Sex			
Female*	—	—	
Male	1.26	1.19, 1.34	<0.001
Age at baseline	1.01	1.01, 1.02	<0.001
Age range at baseline			
> 65 years*	—	—	
18–25 years	0.67	0.58, 0.77	<0.001
26–35 years	0.58	0.52, 0.66	<0.001
36–45 years	0.55	0.50, 0.61	<0.001
46–55 years	0.57	0.52, 0.62	<0.001
56–65 years	0.69	0.64, 0.75	<0.001
Self-declared skin color			
Asian*	—	—	
Black	1.45	0.90, 2.33	0.13
Indigenous	1.28	0.17, 9.45	0.81
Brown	1.47	0.96, 2.23	0.073
White	1.38	0.91, 2.08	0.13
Unknown	2.20	1.46, 3.31	<0.001
Residence region at baseline			
Midwest*	—	—	
Northeast	1.00	0.85, 1.17	0.98
North	0.92	0.72, 1.19	0.53
Southeast	0.97	0.84, 1.12	0.67
South	1.11	0.96, 1.29	0.17
Cohort entry period			
2000–2003*	—	—	
2004–2007	1.31	1.20, 1.42	<0.001
2008–2011	1.10	1.01, 1.19	0.021
2012–2015	0.83	0.75, 0.93	<0.001
Diagnose ICD10 at baseline			
C82-Follicular non-Hodgkin’s lymphoma*	—	—	
C820-Non-Hodgkin’s lymphoma, small cleaved cells, follicular	0.78	0.54, 1.12	0.18
C821-Non-Hodgkin’s lymphoma, mixed, small and large cleaved cells, follicular	0.72	0.50, 1.05	0.091
C822-Non-Hodgkin’s lymphoma, large cell, follicular	0.71	0.50, 1.03	0.071
C827-Other types of non-Hodgkin lymphoma, follicular	0.86	0.59, 1.24	0.42
C829-Non-Hodgkin’s lymphoma, follicular, unspecified	0.79	0.55, 1.13	0.20
Clinical stage at baseline			
I*	—	—	
II	0.99	0.86, 1.14	0.87
III	1.28	1.13, 1.46	<0.001
IV	1.36	1.19, 1.54	<0.001
Unknown	1.60	1.41, 1.80	<0.001
Tumor grade at baseline			
G1*	—	—	
G2	2.94	1.10, 7.86	0.032
G3	0.84	0.71, 1.00	0.051
GX	1.31	1.20, 1.44	<0.001
Chemotherapy at baseline			
Other Schemes*	—	—	
Rituximab based	0.67	0.55, 0.81	<0.001
Unknown Schemes	1.42	1.33, 1.52	<0.001
Frailty index at baseline	1.00	1.00, 1.00	0.005
Comorbidity Charlson Index at baseline	1.06	1.05, 1.07	<0.001
Time in the cohort	0.95	0.95, 0.95	<0.001

(Armitage et al., 2017) HR, hazard ratio; CI, confidence interval.

baseline comparator (reference category).

Therapeutic schemes from years 2000–2007, are not registered in the databases.

**FIGURE 5:**
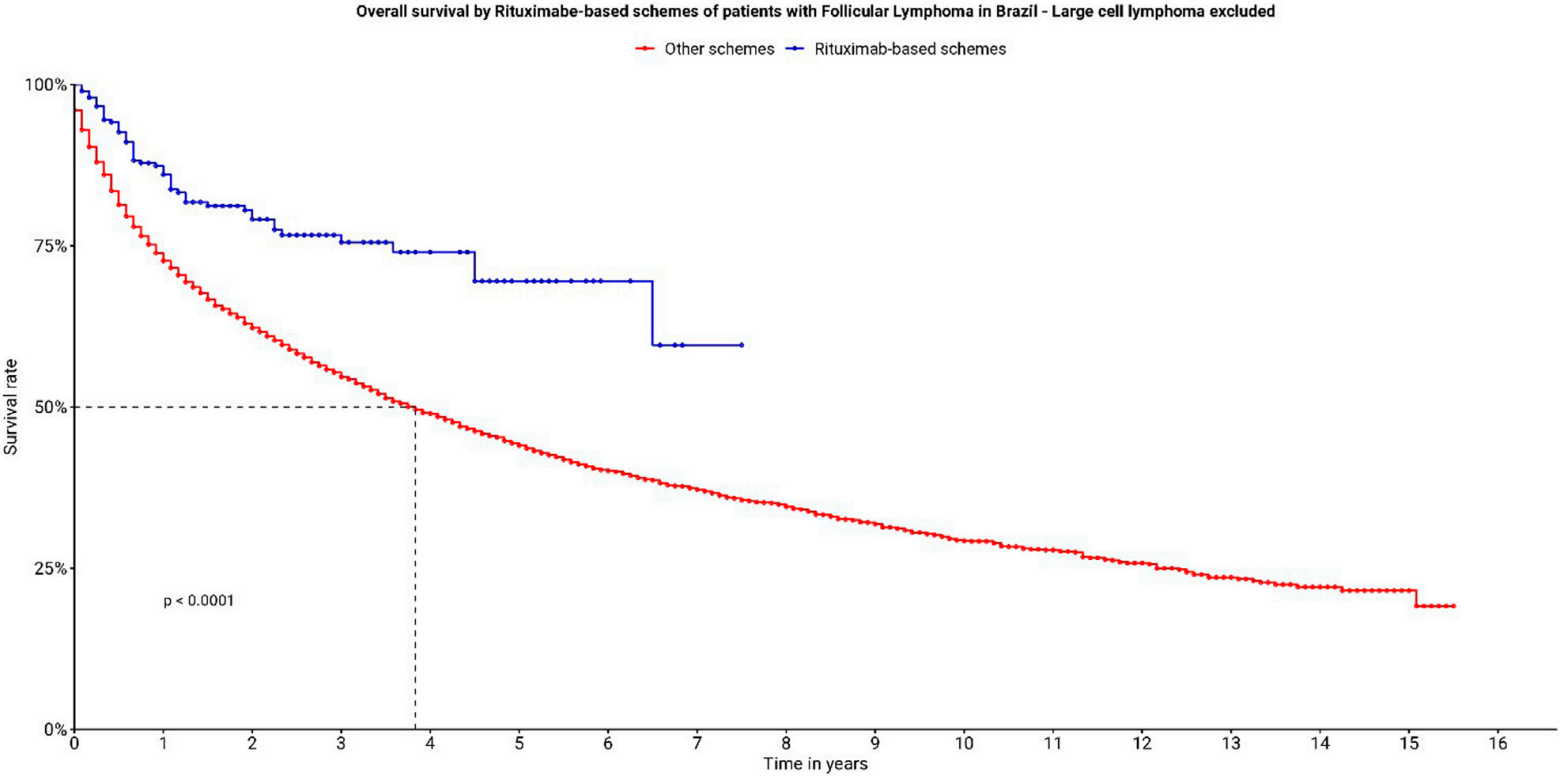
Overall survival by Rituximab-based schemes of patients with follicular lymphoma in Brazil—large cell lymphoma excluded.

Regarding the sensitivity analysis performed, with the exclusion of 1900 patients with ICD C822, some changes occurred in the frequency distribution, but they were not sufficient to modify either the direction or the strength of the associations observed in the complete cohort (Tables 3, 4) and (Figure 5).

**TABLE 3:** Epidemiological characteristics in sensitivity analysis excluding patients with ICD C822.

Patients Characteristics	N = 8,109
Sex	
Male	4,285 (53%)
Female	3,824 (47%)
Age at baseline	55 (16)
Age range at baseline	
>65 years	2,366 (29%)
56–65 years	1,961 (24%)
46–55 years	1,656 (20%)
36–45 years	1,012 (12%)
26–35 years	673 (8.3%)
18–25 years	441 (5.4%)
Self-declared skin color	
Unknown	4,290 (53%)
White	2,822 (35%)
Non-Caucasian (Mixed)	802 (9.9%)
Black	125 (1.5%)
Asian	67 (0.8%)
Indigenous	3 (<0.1%)
Residence region at baseline	
Southeast	3,974 (49%)
South	2,182 (27%)
Northeast	1,399 (17%)
Midwest	368 (4.5%)
North	186 (2.3%)
Cohort entry period	
2008–2011	2,446 (30%)
2012–2015	1,936 (24%)
2000–2003	1,929 (24%)
2004–2007	1,798 (22%)
Diagnose ICD10 at baseline	
C829-Non-Hodgkin’s lymphoma, follicular, unspecified	3,762 (46%)
C820-Non-Hodgkin’s lymphoma, small cleaved cells, follicular	2,865 (35%)
C827-Other types of non-Hodgkin lymphoma, follicular	792 (9.8%)
C821-Non-Hodgkin’s lymphoma, mixed, small and large cleaved cells, follicular	641 (7.9%)
C82-Follicular non-Hodgkin’s lymphoma	49 (0.6%)
Clinical stage at baseline	
Unknown	3,337 (41%)
IV	1,678 (21%)
III	1,466 (18%)
II	1,043 (13%)
I	585 (7.2%)
Tumor grade at baseline	
#xa0; GX	6,675 (82%)
G1	978 (12%)
G3	452 (5.6%)
G2	4 (<0.1%)
Chemotherapy at baseline	
Unknow Schemes*	5,484 (68%)
Other Schemes	2,322 (29%)
Rituxumab based	303 (3.7%)
Frailty index at baseline	14 (166)
Comorbidity Charlson Index at baseline	4 (3)
Time in the cohort	31 (37)
Event type	
Censuring	4,387 (54%)
Death	3,722 (46%)

*(Armitage et al., 2017) n (%); Mean (SD).

Therapeutic schemes from years 2000–2007, are not registered in the databases.

**TABLE 4:** Univariate analysis of patient characteristics on survival in sensitivity analysis excluding patients with ICD C822.

Patients Characteristics (8,109)	HR	95% CI	*p*-value
Sex			
Female*	—	—	
Male	1,24	1.16, 1.33	<0.001
Age at baseline	1,01	1.01, 1.02	<0.001
Age range at baseline			
>65 years*	—	—	
18–25 years	0,68	0.58, 0.80	<0.001
26–35 years	0,59	0.52, 0.67	<0.001
36–45 years	0,56	0.50, 0.63	<0.001
46–55 years	0,59	0.54, 0.65	<0.001
56–65 years	0,7	0.64, 0.76	<0.001
Self-declared skin color			
Asian*	—	—	
Black	1,17	0.70, 1.94	0,55
Brown	1,29	0.84, 1.99	0,25
Unknown	2	1.32, 3.05	0,001
White	1,22	0.80, 1.87	0,35
Residence region at baseline			
Midwest*	—	—	
Northeast	1,05	0.88, 1.26	0,56
North	0,96	0.72, 1.27	0,76
Southeast	1,03	0.87, 1.21	0,77
South	1,2	1.01, 1.42	0,035
Cohort entry period			
2000–2003*	—	—	
2004–2007	1,31	1.20, 1.43	<0.001
2008–2011	1,14	1.04, 1.24	0,004
2012–2015	0,82	0.73, 0.93	0,001
Diagnose ICD10 at baseline			
C82-Follicular non-Hodgkin’s lymphoma*	—	—	
C820-Non-Hodgkin’s lymphoma, small cleaved cells, follicular	0,78	0.54, 1.12	0,18
C821-Non-Hodgkin’s lymphoma, mixed, small and large cleaved cells, follicular	0,72	0.50, 1.05	0,09
C827-Other types of non-Hodgkin lymphoma, follicular	0,86	0.59, 1.25	0,42
C829-Non-Hodgkin’s lymphoma, follicular, unspecified	0,79	0.55, 1.13	0,2
Clinical stage at baseline			
I*	—	—	
II	1,05	0.89, 1.23	0,57
III	1,33	1.15, 1.54	<0.001
IV	1,41	1.22, 1.63	<0.001
Unknown	1,69	1.48, 1.94	<0.001
Tumor grade at baseline			
G1*	—	—	
G2	2,97	1.11, 7.96	0,03
G3	0,75	0.61, 0.92	0,005
GX	1,34	1.20, 1.50	<0.001
Chemotherapy at baseline			
Other Schemes*	—	—	
Rituximab based	0,62	0.47, 0.80	<0.001
Unknown Schemes	1,51	1.39, 1.63	<0.001
Frailty index at baseline	1	1.00, 1.00	0,008
Comorbidity Charlson Index at baseline	1,07	1.05, 1.08	<0.001
Time in the cohort	0,95	0.95, 0.95	<0.001

^1^HR, hazard ratio; CI, confidence interval.

*baseline comparator (reference category).

Therapeutic schemes from years 2000–2007, are not registered in the databases.

### 3.2 Therapeutic regimens

Regarding therapeutic regimens, the most used regimen was CHOP (13%), followed by CVP (9.7%) and R-CHOP (3.3%). Four hundred and ninety-eight patients (4.9%) used regimens containing rituximab. Out of the patients included in the cohort, 2,689 used regimens recommended in the Brazilian Ministry of Health’s guideline (Table 5).

**TABLE 5:** Frequent protocols patients in the cohort.

Therapeutic regimens	N (%)
Most used schemes in the cohort	
CHOP	1,283 (13%)
Cyclophosphamide	175 (1.7%)
Cyclophosphamide + Fludarabine	66 (0.7%)
Chlorambucil	305 (3.0%)
Chlorambucil + Prednisone	42 (0.4%)
CVP	966 (9.7%)
R-CHOP	328 (3.3%)
R-CVP	64 (0.6%)
Other Schemes	6,740 (67%)
Schemes recommended by the Brazilian Ministry of Health	
CHOP	1,283 (13%)
CVP	966 (10%)
FCM	8 (<0.1%)
R-CHOP	328 (3.3%)
R-CVP	64 (0.6%)
Rituximab	40 (0.4%)
Schemes with rituximab	
Schemes with rituximab	498 (4.9%)
Others Schemes	9,511 (95.1%)

Abbreviations: CHOP: cyclophosphamide, doxorubicin, vincristine, prednisone; CVP: cyclophosphamide, vincristine, and prednisolone; FCM: fludarabine, cyclophosphamide, and mitoxantrone; R-CHOP: Rituximab combined with CHOP; R-CVP: Rituximab combined with CVP.

It is important to emphasize that, as this is a 16-year cohort, changes in treatment patterns over time can be observed. For example, rituximab was only incorporated into the SUS in 2013. Therefore, although it was used before its incorporation, there is no follow-up data for patients using rituximab for more than 8 years in the cohort. This is demonstrated through the survival curves of therapeutic regimens.

**FIGURE 6:**
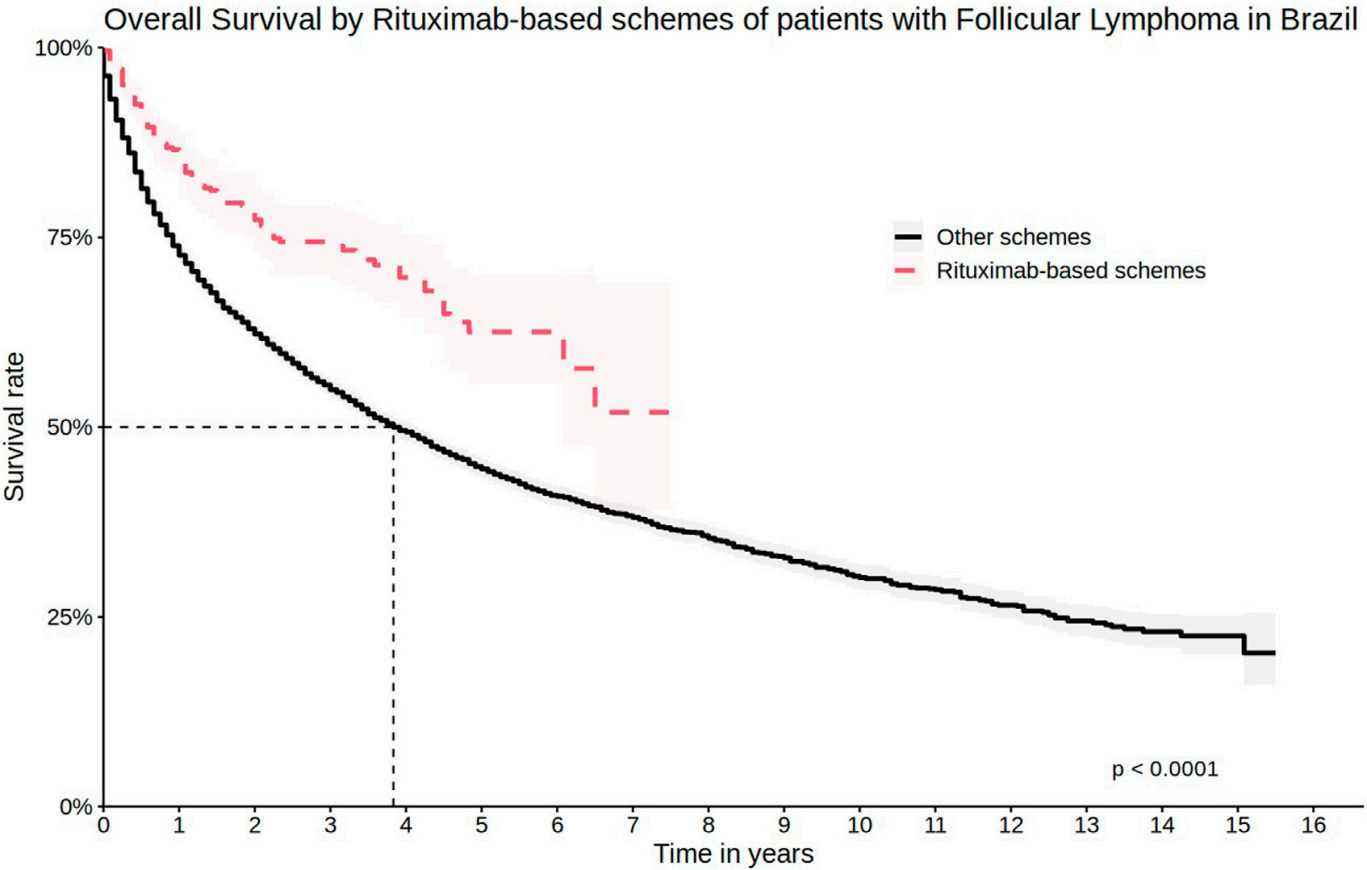
Overall survival by Rituximab-based schemes of patients with Follicular Lymphoma in Brazil.

**FIGURE 7:**
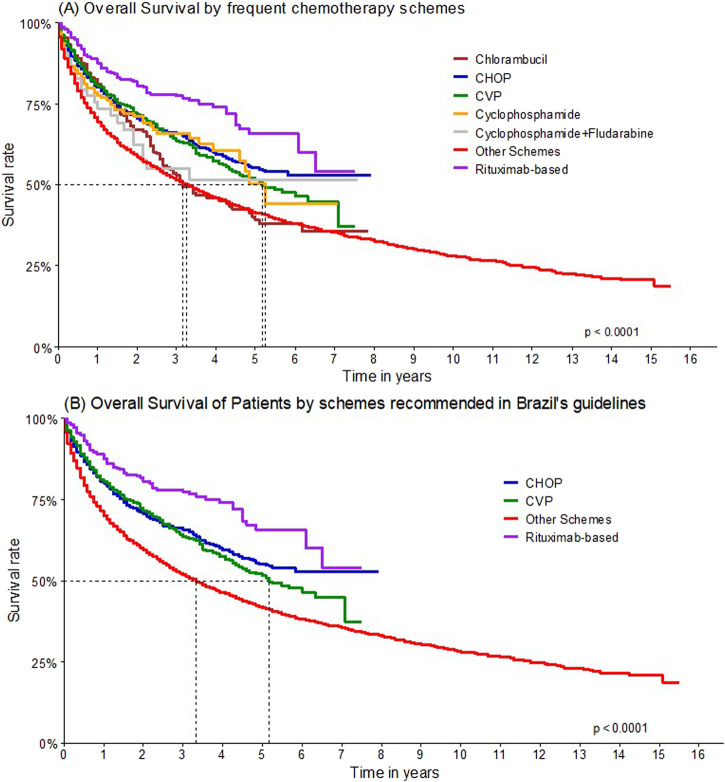
Overall survival by chemotherapy schemes.

The estimated OS considering regimens with rituximab at one and 5 years of follow-up was 85.2% (95% CI 82.0%–88.6%) and 62.5% (95% CI 55.8%–70.0%), respectively (Figure 6). When considering only the regimens mentioned by the Brazilian Ministry of Health’s guideline, and the main protocols used by cohort patients, R-CHOP (88.3%; 95% CI 84.6%–92.1%) and R-CVP (82.1%; 95% CI 72.5%–92.9%) were the regimens with the highest 1-year survival probability (Figure 7).

Univariate analyses to assess the impact of therapeutic regimens on patient survival were conducted, and it was shown that regimens not associated with rituximab are associated with lower survival rates (Table 6).

**TABLE 6:** Univariate analysis by therapeutic schemes.

Therapeutic schemes	Hazard ratio (95%CI)	*p* Value
Most used schemes in the cohort		
CHOP*	-	-
Cyclophosphamide	1.08 (0.82–1.43)	0.57
Cyclophosphamide + Fludarabine	1.30 (0.87–1.94)	0.21
Chlorambucil	1.33 (1.10–1.62)	0.004
Chlorambucil + Prednisone	1.16 (0.70–1.91)	0.56
CVP	1.05 (0.91–1.21)	0.50
R-CHOP	0.64 (0.50–0.83)	<0.001
R-CVP	0.69 (0.40–1.20)	<0.001
Other Schemes	1.55 (1.40–1.72)	<0.001
Schemes with rituximab		
Schemes with rituximab*	-	-
Others Schemes	1.50 (1.24–1.81)	<0.001

Abbreviations: CHOP: cyclophosphamide, doxorubicin, vincristine, prednisone; CVP: cyclophosphamide, vincristine, and prednisolone; FCM: fludarabine, cyclophosphamide, and mitoxantrone; R-CHOP: Rituximab combined with CHOP; R-CVP: Rituximab combined with CVP.

baseline comparator (reference category).

## 4 Discussion

This is the largest study with epidemiological and clinical aspects of LF in Brazil, with data from all Brazilian regions, spanning a long period (16 years). While there are similar international studies that compile health data to understand the profile of FL patients worldwide, such as those conducted in Spain and the Netherlands, it is important to explore the available data in Brazil to plan government actions, for example, through health technology assessment. Since more than 70% of the Brazilian population depends on public health services for oncological treatments (Dinnessen et al., 2021; Mozas et al., 2020).

Male patients and those over 65 years of age had worse prognostic predictors. The epidemiological profile of Brazilian FL patients appeared like that described by other international cohorts. In the USA, Batlevi and colleagues (2020) describe their patients as distributed almost evenly between men and women, with an average age of 57 years (Batlevi et al., 2020). In China, Zha and colleagues (2021) described the FL population with a slight female trend and an average age at diagnosis of 53 years (Zha et al., 2021). However, Lim and colleagues (2020), in an analysis of FL patients in Singapore, identified 67.9% of men diagnosed with FL with an average age of 59 years (Lim et al., 2020).

Regarding most patients being residents of the Southeast or South regions compared to other regions, this high number of FL patients treated by the SUS and a higher number of deaths may be correlated with the population size. Additionally, demographics, socioeconomic factors, and the structure of healthcare services can also explain the differences among Brazilian states. A recent study showed that the Human Development Index (HDI), income inequality (Gini index), and the rate of hospital beds per thousand inhabitants in Brazilian states are associated with the respective morbidity and mortality due to NHL. In this study, Brazilian states with higher HDI, lower Gini index, and higher hospital bed rates per thousand inhabitants had a lower mortality/incidence ratio for NHL (survival estimate) (Caló et al., 2022).

When comparing the overall survival curves with other countries, a small difference in five and 10 years between Brazilian FL patients and American patients was observed. After five and 10 years of diagnosis, it is expected that approximately 92% and 80% of FL patients in the USA have survived, respectively, compared to 45.3% and 30.7% in Brazil. In the same study, the median overall survival was not reached, while in Brazil, the median overall survival was 49 months (Batlevi et al., 2020). This result may be entirely related to the therapeutic management of patients. In the American study, most patients received rituximab-containing regimens (52.1% of patients in the first line) - while in Brazil, only 5.0% of patients used any rituximab-containing regimen during the cohort. As shown in the univariate analysis, in Brazil, patients using rituximab have higher survival rates when compared to regimens without rituximab. The progressive decrease in survival of Brazilian FL patients may also be related to the fact that most patients underwent their first treatment after the age of 65. Furthermore, in the present cohort, the entry date was defined as the first procedure, which necessarily consists of systemic treatment. This may result in the exclusion of a significant period between diagnosis and initiation of treatment for patients who did not receive any therapy at the time of diagnosis or who underwent radiotherapy alone (e.g., for cases of localized disease). Therefore, survival data in this cohort reflect survival after chemotherapy administration, which may, in part, explain the discrepancies observed compared to cohorts from other countries.

We did not find data to compare the survival rate of the Brazilian population by gender with other populations. However, the worst results were observed in the Brazilian male population. Regarding staging and histological grade, perhaps the absence of this information in many patients results from underreporting. Due to the high number of treated patients, it may have been difficult to collect accurate information about staging and histological grade in the medical records of the SIA, where this information is mandatory. In the absence of this data, it is not possible to make a proper comparison of the survival rate of Brazilian data with other populations. However, a study conducted with 1,088 American patients showed that the 5-year survival rate is 97% for stage I patients, 91% for stage II, 92% for stage III, and 88% for stage IV (Batlevi et al., 2020).

The absence of histopathological and molecular data in the database made it difficult to assess the histopathological transformation of FL into more aggressive NHL. Using the change in ICD as a proxy for transformation may not reflect the reality of the Brazilian population and may be the result of an inaccurate diagnosis. Nevertheless, it is important to note that histopathological transformation occurs in about 20% of patients during the disease (Batlevi et al., 2020; Tsimberidou and Keating, 2005).

In terms of treatment, Brazilians seem to be in line with the practices of major treatment protocols. It is important to note that FL is biologically and clinically heterogeneous, and there is no established single standard treatment, which presents challenges in its clinical management. The current guidelines from the National Comprehensive Cancer Network on B-cell lymphomas endorse CHOP, CVP in combination with rituximab as the preferred immunochemotherapy regimen. The guidelines also support optional maintenance with rituximab. The introduction of first line chemoimmunotherapy, employing a combination of chemotherapy agents and the anti-CD20 monoclonal antibody, such as R-CHOP, has led to an overall response rate of over 90% among first-line FL patients (Flinn et al., 2014).

When evaluating the therapeutic regimens used by patients in the cohort, most received CHOP, CVP, and R-CHOP. However, 67% of patients were treated with distinct regimens, demonstrating a lack of a well-defined treatment standard. The same occurred when stratifying patients according to the regimens recommended by the DDT. A study conducted with first-line FL patients in Latin America demonstrated that most patients received chemoimmunotherapy (94.5%). Approximately 70% received CHOP-like regimens combined with rituximab: R-CHOP (51.3%) or RCVP (17.6%). Another 19.2% of patients received only CHOP or CVP (Pavlovsky et al., 2022).

The results of the present observational studysuggest that the use of rituximab can significantly improve the survival of patients with FL compared to therapy without rituximab, like other studies. A German study demonstrated that adding rituximab to first-line chemotherapy with CHOP significantly improved the OS of patients compared to chemotherapy alone (Hiddemann et al., 2005). Another German study also showed that the use of rituximab in combination with first-line chemotherapy followed by maintenance with interferon prolonged the overall survival of patients (Herold et al., 2007). The study by Federico and colleagues (2000) developed a prognostic model for FL that included the use of rituximab as a positive factor in improving OS (Federico et al., 2013). Overall, studies evaluating the OS of FL patients treated with rituximab have shown consistent results. It has been observed that rituximab treatment, whether in combination with chemotherapy or as monotherapy, significantly improves the OS of FL patients. Some studies have also highlighted that patient age, the presence of comorbidities, and the stage of FL at the time of diagnosis can affect OS and should be considered in treatment decisions. In general, the results suggest that rituximab remains an important and effective option in the treatment of FL, even with the wide range of therapeutic options available (Hiddemann et al., 2005; Junlén et al., 2015; Marcus et al., 2005; Shi et al., 2017; Swenson et al., 2005; Tan et al., 2013).

The new treatments available for FL offer options for patients who do not respond to or develop resistance to the rituximab and chemotherapy regimen. Among these new options are targeted therapies such as ibrutinib, which has shown efficacy in treating FL patients, as well as immunotherapies. Another strategy is the combination of different types of treatments, such as chemotherapy and targeted therapies. Recently, studies have evaluated the combination of rituximab with other targeted therapies, such as the PI3K inhibitor idelalisib, and have shown promising results. Additionally, new CAR-T cell-based therapies are also being developed for the treatment of FL.

These new treatments could not be analyzed in this cohort due to its timeframe (2000–2015), as these are technologies that have been introduced to the market more recently. It is important to note that although rituximab was approved by the National Health Surveillance Agency in 2000 and the first Ministry of Health ordinance that included rituximab in the list of medications available in SUS for the treatment of FL was in 2002, it was only in 2013 that the National Commission for the Incorporation of Technologies (CONITEC) in SUS recommended rituximab for the treatment of FL (ANVISA. MABTHERA, 1998). Following this recommendation, the Ministry of Health expanded access to the medication through different stages and clinical protocols. Thus, there was not a single date of rituximab incorporation into SUS, but rather a gradual process of inclusion and expanded access to the medication over the years, based on scientific evidence and patient needs, as evidenced in the cohort in question.

One of the main limitations of this study is the use of an administrative database, which had shortcomings in the recording of clinical information. Additionally, there are challenges in the coding of procedures and a lack of socioeconomic and demographic variables. Direct access to histopathological, molecular, clinical, and therapeutic variables or information on FL subtypes was not possible. Therefore, the diagnostic confirmation was based on the correlation of ICD-10 and the procedures defined as inclusion criteria in the patient registry, resulting in a significant amount of missing data. Regarding ICD-10, we included in the analysis the ICD that in the SUS therapeutic guideline (C82, C821, C822, C827 and C829). Therefore, within these ICD, especially C829, there is the possibility that there are patients with Grade 3B Follicular Lymphoma,an aggressive disease, much more like diffuse large B-cell lymphoma. The presence of these patients could possibly influence the survival outcomes of the overall cohort, making it more heterogeneous. However, with the data available in the cohort, it is not possible to identify whether these patients are present in the database.

Treatment decisions are influenced by prognosis. The lack of data on histological grading and clinical staging in many patients likely occurred due to underreporting. Due to the large number of treated patients, the filling of information in the SIA forms, where this information is mandatory, may not have been consistently performed. Additionally, there is a high percentage of missing data in national oncology databases in Brazil, as reported by other authors, and this issue should be addressed to enable reliable epidemiological analyses in the country (Paulino et al., 2020). Despite that, this is the largest cohort of FL ever recorded in Brazil, and having consolidated epidemiological data is essential for structuring programs that cater to these patients.

A critical consideration emerging from this study is the need for improved data collection practices within Brazilian databases. Currently, limitations in data completeness restrict the scope and precision of epidemiological analyses, which are essential for understanding disease trends, diagnosis, treatment outcomes, and public health implications.

The National Therapeutic Guideline for FL was published in 2014, recommending the use of rituximab either as monotherapy or in combination with other medicines (Ministério da Saúde, 2014). However, oncologies services in Brazilian are financed and payed by treatment procedure, e.g., “c*hemotherapy for intermediate or high-grade non-Hodgkin lymphoma (first Line)”* with a fixed price. It allows physicians and medical services to choose between the different therapeutic regimens approved by ANVISA and available in the Brazilian market. For this reason, this cohort presents data on patients treated with rituximab before its formal incorporation into national health service (SUS). In the instance of Rituximab, an earlier incorporation with adequate funding for oncology services, might have potentially saved lives of patients with this cancer type. This takes into account the potential effects on both the overall survival of patients and the adjusted quality of life years, particularly in comparison to the treatments available within SUS at that specific time.

## Data Availability

The original contributions presented in the study are included in the article/supplementary material, further inquiries can be directed to the corresponding authors.

## References

[B1] Acurcio F. de A. Guerra Junior A. A. da Silva M. R. R. Pereira R. G. Godman B. Bennie M. (2020). Comparative persistence of anti-tumor necrosis factor therapy in ankylosing spondylitis patients: a multicenter international study. Curr. Med. Res. Opin. 36 (4), 677–686. 10.1080/03007995.2020.1722945 31990224

[B2] ANVISA. MABTHERA (1998). Agência Nacional de Vigilância Sanitária. Available at: https://consultas.anvisa.gov.br/#/medicamentos/250000202119750/?substancia=8052.

[B3] Armitage J. O. Gascoyne R. D. Lunning M. A. Cavalli F. (2017). Non-Hodgkin lymphoma. Lancet 390 (10091), 298–310. 10.1016/S0140-6736(16)32407-2 28153383

[B4] Batlevi C. L. Sha F. Alperovich A. Ni A. Smith K. Ying Z. (2020). Follicular lymphoma in the modern era: survival, treatment outcomes, and identification of high-risk subgroups. Blood Cancer J. 10 (7), 74. 10.1038/s41408-020-00340-z 32678074 PMC7366724

[B5] BRASIL. MINISTÉRIO DA SAÚDE (MS) (2014). SECRETARIA DE ATENÇÃO À SAÚDE (SAS). Protocolos clínicos e diretrizes terapêuticas em oncologia. Ministério Saúde.

[B6] Caló R. dos S. Souza R. A. G. de Alves M. R. Carvalho A. E. de Galvão N. D. (2022). Socioeconomic development and colorectal cancer mortality in a state of the Brazilian Legal Amazon from 2005 to 2016. Rev. Bras. Epidemiol. 25 (Suppl. 1). 10.1590/1980-549720220006.supl.1 35766763

[B7] Departamento de Informática em Saúde. Secretaria Executiva M da S. DATASUS.

[B8] Dinnessen M. A. W. Visser O. Tonino S. H. Posthuma E. F. M. Blijlevens N. M. A. Kersten M. J. (2021). Risk of second primary malignancies in patients with follicular lymphoma: a population-based study in The Netherlands, 1989-2018. Blood Cancer J. 11 (11), 179. 10.1038/s41408-021-00574-5 34775466 PMC8590687

[B9] Federico M. Luminari S. Dondi A. Tucci A. Vitolo U. Rigacci L. (2013). R-CVP versus R-CHOP versus R-FM for the initial treatment of patients with advanced-stage follicular lymphoma: results of the FOLL05 trial conducted by the fondazione italiana linfomi. J. Clin. Oncol. 31 (12), 1506–1513. 10.1200/JCO.2012.45.0866 23530110

[B10] Flinn I. W. van der Jagt R. Kahl B. S. Wood P. Hawkins T. E. MacDonald D. (2014). Randomized trial of bendamustine-rituximab or R-CHOP/R-CVP in first-line treatment of indolent NHL or MCL: the BRIGHT study. Blood 123 (19), 2944–2952. 10.1182/blood-2013-11-531327 24591201 PMC4260975

[B11] Guerra-Júnior A. A. Pires de Lemos L. L. Godman B. Bennie M. Osorio-de-Castro C. G. S. Alvares J. (2017). HEALTH TECHNOLOGY PERFORMANCE ASSESSMENT: REAL-WORLD EVIDENCE FOR PUBLIC HEALTHCARE SUSTAINABILITY. Int. J. Technol. Assess. Health Care 33 (2), 279–287. 10.1017/S0266462317000423 28641588

[B12] Herold M. Haas A. Srock S. Neser S. Al-Ali K. H. Neubauer A. (2007). Rituximab added to first-line mitoxantrone, chlorambucil, and prednisolone chemotherapy followed by interferon maintenance prolongs survival in patients with advanced follicular lymphoma: an east German study group hematology and oncology study. J. Clin. Oncol. 25 (15), 1986–1992. 10.1200/JCO.2006.06.4618 17420513

[B13] Hiddemann W. Kneba M. Dreyling M. Schmitz N. Lengfelder E. Schmits R. (2005). Frontline therapy with rituximab added to the combination of cyclophosphamide, doxorubicin, vincristine, and prednisone (CHOP) significantly improves the outcome for patients with advanced-stage follicular lymphoma compared with therapy with CHOP alone: results of a prospective randomized study of the German Low-Grade Lymphoma Study Group. Blood 106 (12), 3725–3732. 10.1182/blood-2005-01-0016 16123223

[B14] INCA. Estimativa | 2023 Incidência de câncer no Brasil. 2023.

[B15] Junior A. A. G. Pereira R. G. Gurgel E. I. Cherchiglia M. Dias L. V. Ávila J. (2018). Building the national database of health centred on the individual: administrative and epidemiological record linkage - Brazil, 2000-2015. Int. J. Popul. Data Sci. 3 (1), 446. 10.23889/ijpds.v3i1.446 34095519 PMC8142958

[B16] Junlén H. R. Peterson S. Kimby E. Lockmer S. Lindén O. Nilsson-Ehle H. (2015). Follicular lymphoma in Sweden: nationwide improved survival in the rituximab era, particularly in elderly women: a Swedish Lymphoma Registry Study. Leukemia 29 (3), 668–676. 10.1038/leu.2014.251 25151959

[B17] Lemos L. L. P. de Guerra Júnior A. A. Santos M. Magliano C. Diniz I. Souza K. (2018). The assessment for disinvestment of intramuscular interferon beta for relapsing-remitting multiple sclerosis in Brazil. Pharmacoeconomics 36 (2), 161–173. 10.1007/s40273-017-0579-0 29139001 PMC5805817

[B18] Lim R. M. H. Chan N. P. X. Khoo L. P. Cheng C. L. Tan L. Poon E. Y. L. (2020). A clinico-genotypic prognostic index for *de novo* composite diffuse large B-cell lymphoma arising from follicular lymphoma in asian patients treated in the rituximab era. Sci. Rep. 10 (1), 4373. 10.1038/s41598-020-61378-4 32152442 PMC7062756

[B19] Marcus R. Imrie K. Belch A. Cunningham D. Flores E. Catalano J. (2005). CVP chemotherapy plus rituximab compared with CVP as first-line treatment for advanced follicular lymphoma. Blood 105 (4), 1417–1423. 10.1182/blood-2004-08-3175 15494430

[B20] Ministério da Saúde. PORTARIA N^o^ 1051, DE 10 DE OUTUBRO DE 2014. 2014. Available at: https://www.gov.br/saude/pt-br/assuntos/protocolos-clinicos-e-diretrizes-terapeuticas-pcdt/arquivos/2014/ddt_linfomafolicular_10102014.pdf

[B21] Ministério da Saúde (2013). Rituximabe para o tratamento de linfoma não-Hodgkin de células B, folicular. Available at: http://antigo-conitec.saude.gov.br/images/Incorporados/Relatório_RTX_linfomafolicular_55-FINAL.pdf.

[B22] Mozas P. Nadeu F. Rivas-Delgado A. Rivero A. Garrote M. Balagué O. (2020). Patterns of change in treatment, response, and outcome in patients with follicular lymphoma over the last four decades: a single-center experience. Blood Cancer J. 10 (3), 31. 10.1038/s41408-020-0299-0 32139690 PMC7058022

[B23] Paulino E. de Melo A. C. Silva-Filho A. L. Maciel L. de F. Thuler L. C. S. Goss P. (2020). Panorama of gynecologic cancer in Brazil. JCO Glob. Oncol. 6 (6), 1617–1630. 10.1200/GO.20.00099 33108231 PMC7605369

[B24] Pavlovsky M. Cubero D. Agreda-Vásquez G. P. Enrico A. Mela-Osorio M. J. San Sebastián J. A. (2022). Clinical outcomes of patients with B-cell non-hodgkin lymphoma in real-world settings: findings from the hemato-oncology Latin America observational registry study. JCO Glob. Oncol. 8 (8), e2100265. 10.1200/GO.21.00265 35486884 PMC9088238

[B25] Shi Q. Flowers C. R. Hiddemann W. Marcus R. Herold M. Hagenbeek A. (2017). Thirty-month complete response as a surrogate end point in first-line follicular lymphoma therapy: an individual patient-level analysis of multiple randomized trials. J. Clin. Oncol. 35 (5), 552–560. 10.1200/JCO.2016.70.8651 28029309

[B26] Swenson W. T. Wooldridge J. E. Lynch C. F. Forman-Hoffman V. L. Chrischilles E. Link B. K. (2005). Improved survival of follicular lymphoma patients in the United States. J. Clin. Oncol. 23 (22), 5019–5026. 10.1200/JCO.2005.04.503 15983392

[B27] Swerdlow S. H. Campo E. Pileri S. A. Harris N. L. Stein H. Siebert R. (2016). The 2016 revision of the World Health Organization classification of lymphoid neoplasms. Blood 127 (20), 2375–2390. 10.1182/blood-2016-01-643569 26980727 PMC4874220

[B28] Tan D. Horning S. J. Hoppe R. T. Levy R. Rosenberg S. A. Sigal B. M. (2013). Improvements in observed and relative survival in follicular grade 1-2 lymphoma during 4 decades: the Stanford University experience. Blood 122 (6), 981–987. 10.1182/blood-2013-03-491514 23777769 PMC3739040

[B29] Tsimberidou A. M. Keating M. J. (2005). Richter syndrome: biology, incidence, and therapeutic strategies. Cancer 103 (2), 216–228. 10.1002/cncr.20773 15578683

[B30] Zha J. Fan L. Yi S. Yu H. Zheng Z. Xu W. (2021). Clinical features and outcomes of 1845 patients with follicular lymphoma: a real-world multicenter experience in China. J. Hematol. Oncol. 14 (1), 131. 10.1186/s13045-021-01139-6 34425858 PMC8383436

